# Iranian Immigrant Women’s Experiences of Intimate Partner Violence: A Literature Review

**DOI:** 10.1007/s10903-024-01610-9

**Published:** 2024-07-02

**Authors:** Soudabeh Niroomand, Leila Gholizadeh, Kathleen Baird

**Affiliations:** 1 Faculty of Health, University of Technology Sydney, Broadway, PO Box 123, Sydney, NSW 2007 Australia; 2 School of Nursing and Midwifery, Faculty of Health, University of Technology Sydney, Sydney New South Wales, Australia

**Keywords:** Intimate partner violence, Immigration, Women, Iran

## Abstract

Immigrant and refugee women are vulnerable to experiencing intimate partner violence (IPV) due to a range of factors associated with immigration. This study aims to consolidate existing research concerning IPV among Iranian immigrant women and examine its impact on their lives. A comprehensive literature search for articles of any design published in the English language in the past 15 years was performed using Medline, Embase, CINAHL, International Bibliography of the Social Sciences (ProQuest) and PsycINFO databases. The topic of IPV among Iranian immigrant women has been underexplored in research, and only 11 studies were identified that met the inclusion criteria for this topic. The findings from these studies indicate that Iranian immigrant women have experienced different forms of IPV, with psychological IPV being prominent and replacing physical violence. These experiences have had adverse effects on the women’s physical and mental health. The women's experiences of IPV were influenced by various cultural, religious, and individual factors. They predominantly sought informal help rather than accessing formal resources to address their situations. There is a need for rigorous studies to thoroughly investigate IPV among Iranian immigrant and refugee women. Such research is essential for establishing effective strategies that are culturally sensitive to reduce IPV incidents within this population. Moreover, it is essential to enhance IPV awareness among these women and ensure their access to formal resources that are proficient in addressing IPV. This comprehensive approach not only tackles the immediate issue but also fosters a safer environment and promotes long-term wellbeing within this community.

## Introduction

Intimate Partner Violence (IPV) includes acts of psychological, physical, and sexual abuse by men and women towards their romantic partners of the same or opposite sex [1]. IPV can exert severe and devastating effects on a woman’s physical and mental health, with significant implications for maternal and neonatal mortality and morbidity [2, 3]. IPV presents a significant public health challenge affecting diverse demographic groups, including immigrants [4]. Immigration, whether forced or voluntary, creates a stressful environment within families, which can lead to increased tensions and interpersonal relationship issues [5–7]. In addition, sociocultural differences between immigrants’ home countries and the country of arrival can contribute to IPV [8]. Immigrant and refugee women face heightened vulnerability when it comes to IPV, which can include cases of intimate partner murder [9]. Due to factors related to immigration, such as language barriers, shift in gender roles during resettlement, and past experiences of violence in their home country or during immigration, immigrant women have historically been at a higher risk of experiencing IPV, which can lead to a cycle of abuse [10, 11]. Cultural, legal, and institutional barriers, coupled with a lack of strong support networks, the fear of losing custody of their children, and financial concerns, often discourage immigrant women from seeking help and escaping abusive relationships [9, 12]. Refugees, who are compelled to depart their homeland, may be at a greater risk of undergoing significant physical and psychological trauma both prior to, during, and after their migration [7].

Iran, as a Middle Eastern country, has a conservative and patriarchal culture that is reflected in its laws, religion, and social norms. Following the Islamic revolution in Iran in 1979, the legal age for marriage was lowered, requiring girls to obtain paternal consent for their first marriage irrespective of their age [13, 14]. Concurrently, the state enacted significant changes to divorce laws, including the unilateral right of men to divorce, the sanctioning of polygamous marriages for men (permitting up to four wives), and the exclusive authority of men to initiate divorce proceedings [13, 14]. Some viewpoints associate the patriarchal norms with the Persian culture [15]; however, these gender-based obligations could potentially be influenced by Islamic traditional beliefs, which are prevalent in many Middle Eastern countries [16]. Patriarchy and traditional masculinity are prevalent norms in the Middle East, which often result in women being socialized from birth to be submissive and occupy lower positions than men [17, 18]. According to studies in some provinces of Iran, 73–87% of women experienced IPV, sexually, emotionally, or physically [19–22].

In recent decades, there has been an increase in the number of Iranians who have migrated to Western countries due to economic and socio-political instability in Iran [23]. Therefore, the number of Iranian immigrants has seen a growth from half a million before the revolution to 3.1 million in 2019 [24]. Despite the growing interest in studying IPV worldwide, very few studies have specifically examined the experiences of Iranian immigrant women following migration. This knowledge gap has adverse consequences for this particular population, as it hinders their access to essential support services [14]. Therefore, this literature review aims to answer the following research questions: (1) How does immigration affect the nature and characteristics of IPV encountered by Iranian immigrant women? (2) What are the contributing factors to the occurrence of IPV experienced by Iranian immigrant women? 93) How do Iranian immigrant women perceive and respond to IPV? (4) What are the factors that hinder or facilitate Iranian immigrant women in seeking help for IPV?

## Methods

This review employed an integrative review approach, which allows for the inclusion of multiple types of studies. The integrative review is a systematic and comprehensive research methodology that is used to analyse and synthesise existing literature on a specific research topic [25]. The method of this review followed six phases: First, the problem was identified, and research questions were formulated. Second, inclusion and exclusion criteria were defined. Third, the specific information to be extracted from the studies was determined. Fourth, the included studies underwent evaluation. Fifth, the results were interpreted within the existing body of knowledge and then presented in the form of this review. To commence the search, a block-building strategy was adopted across five databases. During the study selection process, we adhered to the PRISMA (Preferred Reporting Items for Systematic Reviews and Meta-Analyses) guidelines, culminating in a total of 11 studies for analysis [26].

### Search Strategy

The search for the relevant literature covered multiple databases, including Medline, Embase, CINAHL, International Bibliography of the Social Sciences (ProQuest) and PsycINFO. Search terms and keywords were “Domestic violence”, “Domestic violence against women”, “Family violence”, “Intimate partner violence”, “Battered women”, “Wife beat*”, “Gender-Based Violence”, “Dating Violence”, “Domestic abuse”, “Family abuse”, “Intimate partner abuse”, “Partner Violence”, “Victim”, “Immigrant*”, “Immigra*”, “Emigra*”, “Refugee*”, “Asylum seek*”, “Women”, “woman”, “female*”, “Iran*”, “Iranian*”, “Persian*”, and “Farsi”.

### Inclusion and Exclusion Criteria

This review focused on research articles that investigated domestic violence among Iranian immigrant women. The inclusion criteria included peer reviewed papers published in English and published within the last 15 years. Studies of any design involving Iranian women leaving their country for any reason, voluntary or forced, were selected to provide a more comprehensive perspective. To ensure a comprehensive review, the references and citations of each article were also screened for any possible additional relevant articles. The exclusion criteria encompassed studies that focused on any other kind of domestic violence against women except IPV, men, children, and women who were not of Iranian origin. Additionally, publications that were not electronically published such as books, reports, conference papers, working papers, editorials, websites, and documents were excluded.

### Screening

**Fig. 1:**
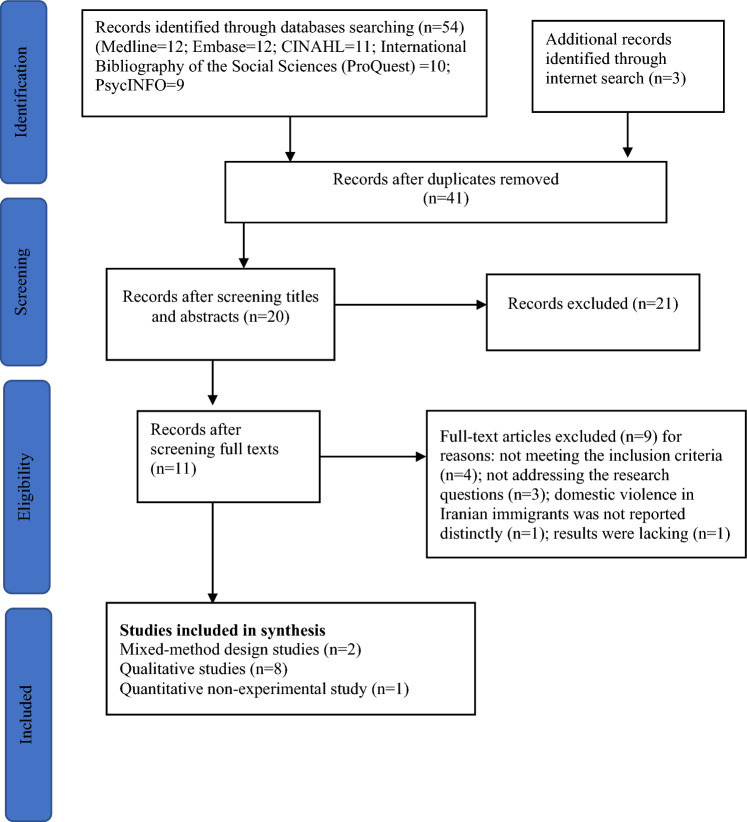
PRISMA diagram depicting the flow of studies from identification to inclusion

A total of 54 articles were initially retrieved from five databases. The databases included Medline, Embase, CINAHL, ProQuest, and PsycINFO. An additional three articles were identified through an internet search. We initially imported the articles into an EndNote library. After removing 13 duplicates, 41 articles underwent a screening process of review. The titles and abstracts were checked to ensure they met the eligibility criteria. During this process, 21 articles were excluded. The full texts of the remaining 20 articles were reviewed, leading to a further exclusion of 9 papers. 11 articles met the inclusion criteria and were included in the review (Fig. 1). Two researchers (Leila Gholizadeh and Soudabeh Niroomand) completed the article selection process independently, and any inconsistencies were resolved by further discussion (Table 1).

**Table 1:** Summary of Included Peer-reviewed Articles Based on MMAT

Author (year) Country	Aim of Study	Type of Study	Study Sample	Data Collection Method	Main Results	Quality
Nikparvar and Stith (2021) USA	-To explore the experience of therapists in their work with Iranian-immigrant IPV clients -To add to the understanding of IPV grounded in the Iranian immigrant culture and ultimately to contribute to a culturally-based conceptualization of IPV among Iranian immigrants -To sensitize therapists regarding culturally appropriate interventions that reflect the concerns of the Iranian immigrant community living in the U.S	Qualitative	N = 8 therapists who have worked with Iranian-immigrant IPV clients	In-depth interview using telephone or skype	-Clients may not recognize IPV if cultural norms and language to describe it are lacking in their country, leading them to live with violence without recognizing it as a problem -A view that anger and violence does not need to be addressed, as it is a common behaviour of men -Many clients felt a sense of disempowerment and easily fell into the role of victim -Clients do not disclose IPV due to a sense of obligation -Clients’ fear of consequences of disclosing -The services are not sufficient/ not proportional	High
Shirpak Maticka-Tyndale Chinichian (2011) Canada	To examine the challenges to gendered marital roles and relationships experienced with immigration to Canada and how this sample of immigrants responded to these challenges	Qualitative	N = 30 Iranian immigrants (15 men and 15 women)	In-depth interviews	-Immigration increases depression, anxiety, and conflict in Iranian families -Difficulty in accepting the freedom that their wives had to dress, socialize, and make decisions for themselves -Women faced challenges entering labour force, e.g., need for re-certification, perception of employers favouring single women with no children, and long work hours -Both men and women's job search caused emotional and financial stress, straining marital relationships -Participants attributed their expectations and sensibilities about acceptability to Iranian culture, rather than Islam or Shar'ia-based regulations. Their beliefs were shaped by cultural understandings of family, gender relations, and modesty	High
Adhami (2016) USA	-To explore domestic violence experiences of Iranian American women, their coping strategies, and assess the effectiveness of intervention tools used by clinicians for this community -To better understand the impact of Iranian culture, immigration, and acculturation on domestic violence within the immigrant Iranian community	Mixed method	Interviews (N = 4) potential Clinical Dissertation Chairs + presentation to a group of (N = 15) mental health providers	Interview + questionnaire	-Low education, self-esteem, poverty, traditional upbringing, family history of violence, childhood sexual abuse, and low acculturation are risk factors for becoming a victim of IPV -Women from minority groups with limited support systems and unhealthy childhood attachments are identified as being vulnerable to staying in an abusive intimate relationship -Immigration and acculturation may have led to a shift in Iranian family structure, with Iranian Americans placing more value on individual freedom and interests. However, community status and pride still likely take priority over individual needs -Immigrant women face greater barriers to leaving abusive relationships compared to non-immigrant women, including feeling more controlled by their abuser, greater fear, social isolation, and legal commitment	High

Author (year) Country	Aim of Study	Type of Study	Study Sample	Data Collection Method	Main Results	Quality
Kian (2017) Canada	-To describe the types of violence women, confront before immigration and explain how power structure and cultural beliefs provoke violence against women -To illustrate whether the violence remains in the same force as before immigration or if there are changes	Qualitative	N = 3 Iranian refugee women who were survivors of IPV and have experienced IPV before and after immigration	Semi-structured interviews	-Physical and sexual violence transformed into psychological violence after immigration, with insults, humiliation, comparison with other women as well as financial violence such as extreme financial dependence to their husbands after immigration -Women's unawareness of immigration regulations, their legal status and regulations related to IPV -The ability to find a job and decreasing financial dependence helped with reducing violence against women after immigration -The woman's long hours or work shifts on weekends led to husband's dissatisfaction and aggression and increasing IPV after immigration	High
Emamianrostami (2022) Sweden	To determine whether socio-cultural adaptation and childhood abuse victimization history are determinants of the victimisation of intimate partner's emotional and economic violence against Persian refugee women in Sweden	Qualitative	N = 36 women	Questionnaires	-The history of childhood abuse victimisation was a significant factor in IPV -The family, society, environment, or the cultural beliefs and norms showed statistically significant effect on of the experience of emotional and economic violence -Socio-cultural adaptations were not significant to IPV	Medium
Guruge, Roche, and Catallo (2012) Canada	To describe the trends in violence throughout the lifespans of women who came to Canada as immigrants or refugees and the resulting physical and mental health symptom patterns	cross-sectional survey	N = 60 women participants from the Iranian and Sri Lankan Tamil communities in Toronto (30 Iranian)	Brief Symptom Inventory (BSI) and the Harvard Trauma Questionnaire (HTQ)	-The most reported actions were insulting, criticizing, forced sexual intercourse, slapping, and pushing -Despite the role of stigma and other barriers, the data indicate considerable rates of IPV, particularly during the most recent phases of their migration history -No significant associations between violence and mental and physical health sequelae among the Iranian -The study found a high prevalence of mental and physical health symptoms in women who had experienced various forms of violence (before, during, and after migration) -Women exposed to violence tended to be between 31–40 years, skilled in speaking English, and currently married with 1–3 children	Medium
Shiraj (2020) The UK	To understand how Iranian women in the UK perceive IPA by men towards women, and what influences such perceptions	Explanatory sequential mixed method design	N = 461 in phase one questionnaires (n = 239 Iranian women) + N = 16 Iranian women in phase two (interviews)	Questionnaires + Structured interviews	-Marital status, education, religion, income, and ethnicity can predict the perception of IPA among Iranian and non-Iranian women in the UK - Iranian women in the UK have better legal knowledge of IPA but are more accepting of male violence compared to non-Iranian women -Iranian women who endorsed the traditional gender roles women tend to believe that IPV is not a significant issue, or they might blame victims for the violence they experience	High

Author (year) Country	Aim of Study	Type of Study	Study Sample	Data Collection Method	Main Results	Quality
Karamali (2021) The UK	-To explore the experiences of Iranian immigrant women who have been subjected to DV within heterosexual relationships -To explore these women’s experiences of therapeutic interventions and other support designed for women who have experienced DV -To explore how women’s sense-making may have changed following a move to the UK	Qualitative	N = 6 Iranian immigrant women who had experienced DV in Iran and the UK	In-depth interviews	-All participants believed they had to maintain a caring role in their family to keep it together, in line with Iranian gender roles, even in cases of IPA -All participants described growing confidence in their ability to make important decisions about their lives as well as a change in their perspectives on gender roles after moving to the UK -All Participants discussed their use of both the UK and Iranian legal systems and utilization of UK counselling services. They expressed the contrast between viewing IPV as a family matter in Iran versus contacting police in the UK, and their experiences with UK counselling services	High
Cochrane and Wolff (2022) Australia	-To understand women’s decisions to undertake the dangers of irregular migration -To highlight how family violence and citizenship can shape women’s decisions to migrate	Qualitative	N = 19 (8 Iranian women and 11 Afghan women)	Interviews	-Women in violent situations often have limited legal and political protections, which further restricts their lives. Despite the dangers, these women take control of their circumstances and migrate in search of safety and citizenship -Many refugee and asylum-seeking women stay in unsafe countries until they experience family violence, which motivates them to undertake irregular journeys as legal routes are often unavailable -Despite border security measures aimed at deterring irregular migration, asylum seekers still undertake perilous boat journeys, risking their lives and potential detention, in pursuit of safety	High
Ghafournia, and Easteal, (2021) Australia	Aims to contribute to the literature on help seeking by looking at what has been found concerning immigrant DV survivors and complementing that with interview material	Qualitative	N = 14 Muslim immigrants (5 Iranian women)	Interviews	-Muslim identity was not seen as a key factor in their experiences of abuse or help-seeking behaviours. Some religious leaders were reported to encourage women to tolerate abuse, but spirituality and religion were seen as empowering forces to respond to abuse -Some victims delayed or did not seek formal help due to concerns that their children needed both parents or fear that their partner would take their children as threatened -The victims were not familiar with the resources available in Australia such as shelters, the role of the police, and migration legislation -The women interviewed had experienced the most adverse help seeking in refuges where they felt the presence of racism and its discriminatory consequences	High
Ghafournia (2017) Australia	To understand the role of religion and spirituality in the experiences of domestic violence among a sample of Muslim women	Qualitative	N = 14 Muslim immigrants (5 Iranian women)	Interviews	-The role of spirituality and religion did not have a decisive influence on the occurrence of abuse, and instead, it enabled women to feel empowered	High

### Methodological Quality Review

The researchers conducted a methodological quality assessment of the included papers. In integrative reviews, assessing the quality of studies can be challenging due to the diverse range of methodological designs, which can make the assessment process more complex [25]. The Mixed Methods Appraisal Tool (MMAT) version 2018 was used to assess the quality of the reviewed studies. The MMAT enables the assessment of the methodological rigor of five types of studies, including qualitative research, randomized controlled trials, non-randomized studies, quantitative descriptive studies, and mixed methods studies [27]. Using the MMAT, specific criteria were employed to evaluate the methodological quality of each study type.

Of the 11 studies included in the review, two were assessed as being of medium quality due to a lack of sampling design, while the remaining nine were assessed as strong quality (Table 2). Out of the 11 studies that were included in this review, nine were assessed as being of strong quality [28–36] and two studies were deemed to be of medium quality due to a lack of proper sampling design [37, 38]. None of the studies were excluded from the review (Table 1).

**Table 2:** The MMAT Critical Appraisal Scores

Author & year	Screening question		1. Qualitative					3. Quantitative non- randomized					4. Quantitative descriptive					5. Mixed methods					overall score
	S1	S2	1.1	1.2	1.3	1.4	1.5	3.1	3.2	3.3	3.4	3.5	4.1	4.2	4.3	4.4	4.5	5.1	5.2	5.3	5.4	5.5	
Nikparvar et al. 2021	Yes	Yes	yes	yes	yes	yes	yes																*****
Shirpak et al. 2011	Yes	Yes	Yes	yes	yes	yes	yes																*****
Adhami et al. 2016	Yes	Yes																yes	yes	yes	yes	yes	*****
Kian et al. 2017	Yes	Yes	yes	yes	yes	yes	yes																*****
Emamianrostami et al. 2022	Yes	Yes	yes	yes	Can’t tell	yes	yes																****
Guruge et al. 2012	Yes	Yes											Yes	Yes	Can’t tell	Can’t tell	Yes						***
Shiraj et al. 2020	yes	Yes																yes	yes	yes	yes	yes	*****
Karamali et al. 2021	Yes	Yes	yes	yes	yes	yes	yes																*****
Cochrane et al. 2022	Yes	Yes	yes	yes	yes	yes	yes																*****
Ghafournia, et al. 2021	Yes	Yes	yes	yes	yes	yes	yes																*****
Ghafournia et al. 2017	Yes	Yes	yes	yes	yes	yes	yes																*****

S1: Are there clear research questions?, S2: Do the collected data allow to address the research questions?, 1.1: Is the qualitative approach appropriate to answer the research question?, 1.2: Are the qualitative data collection methods adequate to address the research question?, 1.3: Are the findings adequately derived from the data?, 1.4: Is the interpretation of results sufficiently substantiated by data?, 1.5: Is there coherence between qualitative data sources, collection, analysis and interpretation?, 3.1: Are the participants representative of the target population?, 3.2: Are measurements appropriate regarding both the outcome and intervention (or exposure)?, 3.3: Are there complete outcome data?, 3.4: Are the confounders accounted for in the design and analysis?, 3.5: During the study period, is the intervention administered (or exposure occurred) as intended?, 4.1: Is the sampling strategy relevant to address the research question?, 4.2: Is the sample representative of the target population?, 4.3: Are the measurements appropriate?, 4.4: Is the risk of nonresponse bias low?, 4.5: Is the statistical analysis appropriate to answer the research question?, 5.1: Is there an adequate rationale for using a mixed method design to address the research question?, 5.2: Are the different components of the study effectively integrated to answer the research question?, 5.3: Are the outputs of the integration of qualitative and quantitative components adequately interpreted?, 5.4: Are divergences and inconsistencies between quantitative and qualitative results adequately addressed?, 5.5: Do the different components of the study adhere to the quality criteria of each tradition of the methods involve?

### Data Extraction

A template was utilised to systematically extract the summary of each included article. The following data were collected for each study: author(s), publication year, study location, research objective, study design, participant sample, data collection methodology, key findings, and research quality assessment. To enhance the rigour of the review, the extracted data underwent a comprehensive discussion and analysis involving all authors in a collaborative process. Furthermore, to increase the rigour of the review, the extracted data was discussed and analysed by two members of the research team.

### Findings

The findings of this review are based on 11 studies, including eight qualitative studies and three quantitative studies (one cross-sectional design and two mixed-methods design). The qualitative studies used different data collection methods, seven studies used interviews as method of data collection [29, 31, 32, 34–37], and one study employed storytelling [30]. The studies were conducted in different countries, including the USA (n = 2), Canada (n = 3), Sweden (n = 1), the UK (n = 2), and Australia (n = 3), with sample sizes ranging from 3 to 461. All studies focused on the experiences of women participants who had experienced IPV, while two studies delved into the encounters encountered by therapists who provided services to these women [28, 30].

### Themes

Despite the variations in the countries where the 11 studies were conducted, there were notable commonalities and differences in the findings. The synthesis of the findings is presented in the following five themes: 1. Experience of IPV post immigration, 2. Types of IPV, 3. The impact of IPV, 4. Factors affecting IPV with three sub-themes of cultural factors, religious factors, and individual factors, and 5. Women’s responses to IPV.

## Experience of IPV post immigration

Of the seven studies [28, 29, 31, 32, 34, 37, 38] that addressed the impact of immigration on IPV, four studies [29, 31, 34, 37] reported that immigration had intensified the experience of IPV for Iranian immigrant women. Although one study maintained immigration alone was not the reason for increasing IPV among Iranian immigrant population [28], and two studies [32, 38] did not provide conclusive evidence regarding the impact of immigration on the experience of IPV among Iranian immigrant women.

Some studies found Iranian immigrants experience significant stress after immigration due to associated financial challenges, changes in their social status, loss of support from family and friends, and conflicts in intimate relationships resulting from the partner's new roles after immigration [29, 31, 34, 37]. For example, a study involving Iranian immigrants living in Canada [31] revealed that family conflicts intensified after immigration due to various disparities in the host country, including women's rights to dress as they pleased, make decisions independently, work outside the home, travel alone without needing their husbands' permission, and receive government economic support after leaving the marriage. This implies that adapting to a new country and culture was particularly challenging when it encompassed both geographical and cultural distinctions that were intricately linked to an individual's core identity, self-confidence, and sense of belonging.

However, according to Adhami's study (2015), immigration alone should not be considered as a risk factor for IPV among Iranian American women. The study found that most recent Iranian immigrants who settled in the USA were able to quickly familiarize themselves with the American justice system and gained a reasonable understanding of their rights in a relatively short period of time. This awareness helped to mitigate the potential link between immigration and IPV [28].

It is important to acknowledge the differences in study participants or research settings. For example, unlike other studies that primarily examined the individual experiences of women [29, 31, 34, 37], Adhami's study relied on third-hand accounts from therapists and their insights on the link between immigration and IPV [28].

## Types of IPV

In 10 of the 11 studies [28–30, 32–38] various forms of IPV were examined. The findings consistently revealed that Iranian immigrant women experienced multiple types of domestic violence, including physical, verbal, sexual, emotional, psychological, and financial abuse [28–30, 32–38]. The documented forms of violence included psychological abuse, such as insulting, criticizing, and intimidating behaviours, physical assaults such as slapping, hitting, and shoving, and sexual abuse, such as non-consensual sexual acts and forced participation in demeaning sexual activities [29, 34, 38]. Emamianrostami (2022) reported that psychological and economic abuse were the two forms of violence used initially, with physical or sexual violence following later.

In Adhami’s study, therapists were of an opinion that Iranian women experienced relatively less physical violence than immigrants from other countries [28]. This may be attributed to several factors within Iranian culture, including the high level of education, their low consumption of alcohol and drugs, and the strong fear and shame associated with having a broken family [28]. These findings align with another research that reported a shift in abusive behaviour patterns among Iranian immigrants, specifically from physical and sexual forms to psychological and financial abuse after immigration [29]. Kian further explained that the involvement of the police, reducing/ending relationships with the in-laws, and reduced connections with the patriarchal system as well as access to social support for women post immigration might have played a role in decreasing physical violence I [29].

## The impact of IPV

Ten studies [28–30, 32–38] reported on the adverse effects of IPV. One study found that Iranian women’ past experiences of family violence and their new challenges upon arrival into a new country such as isolation contributed to their poor mental health status, including the experience of depression post immigration [34]. Supporting these findings, Guruge et al. (2012) also reported a significant number of Iranian immigrant women exhibited physical and mental health issues, which could be attributed to the various forms of violence they had endured throughout their lives [38]. Other studies similarly found that women who had experienced abuse were more likely to exhibit a variety of physical, mental, and socio-behavioural problems [28, 38]. Physical symptoms associated with IPV included headaches, fainting, breathing problems, body aches, high blood pressure, and even cancer [28, 38] and psychological outcomes encompassed low self-esteem, perceived diminished beauty, insecurity, depression, stress, feelings of being trapped, shame, guilt, powerlessness, rejection, and detachment from the family and society [28–30, 32, 33, 35, 36, 38].

IPV not only imposed a negative impact on its immediate victims but also had detrimental effects on subsequent generations, perpetuating a cycle of violence and impacting their future prospects [28, 37]. Violence within the family affected the entire family unit, including children who exhibited a range of symptoms such as anxiety, depression, educational impairment, behavioural problems at school, lack of social skills, increased likelihood of engaging in violent behaviours in boys, and increased risk of future victimization in girls [28, 37].

Nevertheless, the study, conducted by Guruge et al. (2012), did not establish a significant correlation between IPV and subsequent mental or physical health among Iranian immigrant women. However, it is important to consider the limitations of this pilot study, and the sample size of only 30 participants which limits the ability of the study to detect significant differences and the utilization of instruments that were not culturally validated.

## Factors Affecting IPV

The studies identified multiple factors that contributed to the experience of IPV by Iranian immigrant women. These factors were categorized into three sub themes of cultural norms [28–33, 35, 36], religious factors [28, 29, 33, 35, 36], and individual factors [28, 29, 37, 38].

### Cultural Factors

Cultural expectations within the Iranian society have played a significant role in shaping the dynamics between men and women, often leading to women's dependence on men and the deprivation of their basic rights [32, 33]. The patriarchal system, which is deeply rooted in cultural norms, has led to the domination of men over women at different stages of life, thereby denying women independence and freedom, and emboldening violence against women [32, 33]. Iran is known for having a family-oriented culture [31]. In Iranian traditional culture, the concept of a "Good woman" is defined by certain expectations, including refraining from pre-marriage sexual activities to protect the family’s honour, fulfilling the needs of her husband as a wife, and dedicating her life to her children as a mother [32, 33, 35, 36]. Shiraj (2020) reported by accepting the patriarchal norms and endorsing traditional gender roles, some Iranian women inadvertently normalized men’s behavioural violence, including sexual aggression and male domination. Some Iranian women held views that violence was in a man’s nature and it was expected that they use their power against women [29, 30, 33]. These views stemmed from living in a patriarchal society where women were predominantly dependent on men to meet their needs and from their social circles, such as the family and friends, who enforced obedience to husbands’ orders and normalized men’s dominant behaviours [29, 30, 33, 37].

However, Adhami discussed that men's dominance over women might not be solely attributed to the culture and Iranian men might have exploited these cultural norms as a means to exert control, compel women into submission, and perpetuate abusive behaviours [28]. It is, therefore, essential to address both cultural factors and individual behaviours in efforts to combat gender-based violence and promote gender equality.

### Religious Factors

Religious factors were also reported in seven of the reviewed articles [28, 29, 31, 33, 35–37]. Five studies [28, 29, 31, 33, 37] reported that Iranian cultural norms were strongly influenced by Islamic values. Certain cultural norms, rooted in Islamic beliefs, contributed to the perpetuation of men's control and dominance over women [28, 29, 33, 37]. Such norms restricted women's freedom and decision-making, reinforcing gender inequalities [29, 33]. Arguments on religious requirements, such as the compulsory wearing of the Hijab and the requirement for women to seek permission from their husbands to be in public places were intensified after immigration to countries where women have greater freedom of dress and employment choices [29, 33].

However, in a study conducted by Ghafournia et al. (2017) involving 14 Muslim immigrant women from Afghanistan, Bangladesh, Bosnia, Iraq, Lebanon, Pakistan, Somali, Syria and Iran living in Australia, despite a strong adherence to patriarchal ideologies and Islamic principles, they perceived Islam as a source of empowerment, emotional and mental well-being, and a means to navigate challenging situations [35]. Religious immigrant women also expressed a sense of comfort in the belief that God was assisting them during difficult times, and as a result, they relied on prayer as their primary means of coping with domestic violence [35].

### Individual Factors

The reviewed studies linked some individual factors to IPV. Three studies [28, 37, 38] highlighted the significance of a past family history of domestic violence in shaping individuals’ experiences of IPV later in life. Adhami (2015) and Emamianrostami (2022) found that women who had received less respect from their families during their childhood were more vulnerable to entering abusive relationships in their adulthood. They also tended to normalize the abusive behaviours of their partners and often remained in abusive relationships [28, 37].

Two studies [28, 38] reported conflicting results on the impact of education and English language proficiency on the experience of IPV. Adhami (2015) found that Iranian immigrant women with a lower education or limited language skills were more susceptible to IPV after immigration. However, Guruge (2012) found that the majority of Iranian abused women were highly educated and fluent in English. The conflicting findings in the studies conducted by Adhami (2015) and Guruge (2012) may be attributed to several factors. Firstly, the studies employed different measurement tools to assess IPV. Adhami (2015) employed interviews (n = 4), while Guruge (2012) utilized questionnaires (n = 60). Secondly, the study populations differed between the two studies. Adhami's findings were based on the experiences of healthcare providers who worked with Iranian immigrant women facing IPV, whereas Guruge (2012) recruited Iranian immigrant and refugee women. Furthermore, it is likely that women with lower education and limited English language proficiency are more vulnerable to IPV, as their partners may perceive them as lacking the capacity to take any action. Conversely, women with higher education and better English language skills may possess a stronger awareness of women's rights in their host countries and engage in discussions or arguments with their partners regarding these rights, potentially leading to an increased incidence of IPV. These factors should be thoroughly investigated in future research.

In addition to the aforementioned factors, working long hours, engaging in night shifts work, or working on weekends were found to increase family conflicts and violence [29, 31].

## Women’s Response to IPV

Iranian immigrant women responded to IPV in various ways. Some opted to tolerate their abusive relationships, while others sought help from various informal and formal sources [28, 30, 32, 33, 35, 36].

Some women preferred seeking support from their social circles, including family members, relatives, or friends instead of turning to formal sources. This preference stemmed from the belief that in Iranian traditional culture, IPV is a private matter that should not be disclosed due to the associated stigma that it can bring to the family [31]. As well as the dominance of men and the overall oppressive culture against Iranian women who divorce. Previous experiences may have affected the women’s attitudes and courage towards seeking formal help. For example, religious women often turned to prayer or sought help from religious leaders who encouraged them to remain in the family and to just accept the situation they found themselves in [30, 35, 36]. Seeking informal help seemed to increase violence within the family, compelling some women to then proceed to formal help seeking, regardless of the fear they had for their own safety or the safety of their children [36].

In addition, women’s perception of IPV affected their responses. For example, some Iranian immigrant women believed only physical abuse was violence, overlooking other forms of abuse, such as financial abuse, sexual abuse, and emotional mistreatment [30]. Nikparvar & Stith (2021) argued that due to growing up in a country with limited vocabulary for IPV in law and cultural norms, Iranian women faced challenges in understanding and defining their experiences. Supporting these findings, a comparative study in the United Kingdom found that Iranian immigrant women exhibited a higher tolerance for violence when compared to native women [33], potentially due to cultural acceptance of men's violent behaviours [30, 33].

Other reasons that hindered Iranian immigrant women from leaving abusive relationships included their pending immigration status, a sense of obligation to the family, limited language proficiency, inadequate job skills, cultural beliefs, concerns about being a single mother, financial constraints, lack of access to social support, stigma surrounding divorce, and reluctance to seek refuge in shelters [28, 30, 32, 33, 35, 36].

Additionally, factors such as a lack of awareness regarding legal rights, laws, and regulations, unfamiliarity with available services, experiences of racism and discrimination, and feeling disempowered and accepting a victim role hindered these women from seeking formal help [30, 35, 36]. Karamali’s study (2021) supported these findings and added that women, as the primary caregivers in the family, often blamed themselves for the occurrence of violence in the relationship.

Karamali's study (2021) found that immigration overall had a positive impact on abused women's self-assurance, leading to changes in their perspectives on gender roles, decision-making, and help-seeking for IPV. Some women reported seeking formal help from institutions, such as the police, courts, and the legal system [29, 30, 32, 33, 35, 36]. Concluding that most women had positive experiences with domestic violence services in their host countries [29, 30, 32, 33, 35, 36]; however, this was not the case for all women, a few women shared instances of racism, discrimination, or having their claims ignored by the police [35, 36]. These experiences may suggest system issues in the IPV services in certain host countries or merely reflect some individual attitudes or practice within these systems.

## Discussion

The aim of this review was to collect and analyze evidence regarding the experiences of IPV among Iranian immigrant women. In addition, the review aimed to understand the impact of these experiences on the women's health and to explore their responses to IPV and help-seeking behaviors. With the exception of four studies [29, 31, 34, 37], which noted an increase in IPV after immigration, other reviewed studies conducted on Iranian immigrants were not able to clearly demonstrate the role of immigration in increasing or decreasing IPV experiences in this population. Although the available evidence suggests that IPV is highly prevalent in Iran [39], there is a lack of solid evidence demonstrating the pervasiveness of IPV among Iranian immigrant women specifically. This gap in the literature highlights the need for future research to explore the extent of IPV among this population. Based on previous studies conducted on diverse immigrant communities, there is evidence to suggest that immigration can increase the incidence of IPV [40–42], with a potential increase of violence up to 93% [43].

The reviewed studies suggested that Iranian immigrant women are confronted with psychological violence as the most frequently reported form of IPV [29, 34, 38], alongside a spectrum of other domestic abuse types, encompassing physical, verbal, sexual, emotional, and financial abuse [28–30, 32–38]. In addition, psychological and economic abuse manifested as early indicators of IPV, with subsequent occurrence of physical or sexual violence [37]. The implication of this finding is that early identification and management of IPV may reduce the likelihood of physical or sexual abuse. However, this necessitates women recognizing psychological and economic abuse as forms of violence. Many Iranian women do not perceive these as violence and tend to normalize these behaviours in men [30]. Moreover, it was observed that psychological and economical violence substituted physical violence after immigration among this particular population [28, 29]. The shift in the form of violence may be attributed to several factors. One possible explanation is the fear of severe punishment for physical violence in host countries, which may deter Iranian immigrant men from engaging in physical violence that leaves visible evidence of their abusive behaviour. Therefore, they may resort to non-physical forms of abuse that are harder to detect and prove [29]. Furthermore, the disconnection from their extended family and the patriarchal culture might have contributed to the change in the pattern of violence [29]. Without the influence and presence of the traditional patriarchal structures, the power dynamics within a relationship may shift, potentially leading to a greater emphasis on psychological and emotional manipulation as a means of control. It is also possible that Iranian women were reluctant to report instances of physical violence due to potential consequences, such as jeopardizing their immigration status. Additionally, psychological or other forms of violence might have been perceived as a less severe form of violence by Iranian men and women, thereby resulting in men continuing to increase psychological abuse [30]. However, psychosocial abuse can have more serious immediate and long term consequences than physical assault [44]. In line with this finding, psychological abuse within intimate relationships has been reported as the most common type of IPV among various groups of immigrants [45], with the prevalence rates ranging from 14.7% among immigrant women residing in Canada [46] to 76% among Iraqi women living in the USA [43]. However, African immigrant women report increasing levels of physical violence post immigration [47, 48], and reduced psychological/emotional abuse by their partners mostly because of unemployment and economic stresses [49]. It is important to continue to research specific types and patterns of IPV experienced by immigrant women to develop targeted interventions and support services. By understanding the dynamics and shifts in the forms of violence, efforts can be made to raise awareness, provide resources, and implement policies that address the unique challenges faced by this population.

The reviewed studies highlighted a complex interplay of various factors that influenced the experience of IPV among Iranian immigrant women, including individual, ideological, religious, and cultural factors. The role that a patriarchal culture and system of IPV was particularly dominant in the literature. The patriarchal culture, religion, laws, and legislations perpetuated gender inequality and power imbalances, and shaped the relationships between men and women, and their expectations, and social norms in Iran [50, 51]. These laws and norms were found to prioritize male authority and control, while reinforcing traditional gender roles and expectations [52, 53]. The patriarchal system, combined with cultural and religious beliefs and attitudes contributed to the tolerance or acceptance of violence against women [54], normalizing abusive behaviours, make it challenging for women to identify IPV and seek help [55].

Iranian immigrant women used a range of formal and informant strategies to seek help for IPV. However, their families often served as the primary source of advice for a variety of reasons, including the associated stigma, concerns about immigration status, cultural and religious perceptions regarding IPV, language barriers, and a lack of awareness about support services [28, 30–33, 36]. Furthermore, negative past experiences with formal help-seeking in their home country may have contributed to Iranian immigrant women's reliance on their families for support. The domestic violence services available in Iran are often insufficient to protect abused women, [12, 29, 30, 32, 34, 36, 56], and the laws and legislations are commonly more supportive towards men [56]. For example, women may choose to remain in abusive relationships because of the fear of losing their custodianship of their children or due to financial insecurity after divorce [12].

Religious women tended to utilize their spiritual beliefs such as praying, as a source of resilience and security in the face of IPV instead of seeking formal help [35, 36], or they sought advice from clergies who often encouraged them to tolerate the situation [35]. However, staying in an abusive relationship often led to women being subjected to severe abuse [42].

After immigration, the women’s awareness of their civil and legal rights increased, and some women gained self-confidence to seek formal help. Women, who utilized legal services in their host countries, reported an overall positive experience [30, 32]; however, some women reported instances of racism, discrimination or having their cases ignored [36, 42]. These negative experiences further compound the challenges faced by immigrant women seeking formal help and access to justice. Instances of racism and discrimination within a legal system can be indicative of system issues or may be attributed to individual behaviours and practices.

Creating a supportive environment for immigrant women involves developing an understanding of their cultural backgrounds and cultivating an inclusive culture that embraces immigrants with diverse cultures and experiences. Additionally, it is important to conduct a thorough investigation to uncover the root causes and factors contributing to IPV among immigrant populations, as well as the barriers they face when seeking help. This analysis will aid in identifying effective interventions and strategies to support immigrants, including Iranian women.

## Strengths and Limitations of Review

The strength of this review study is evident in its systematic approach and recognized methodology, which contribute to a deeper conceptual understanding of IPV among Iranian immigrant women. Further, two researchers were independently involved in literature search and article selection. One researcher extracted the data from the reviewed studies, and another researcher randomly audited the data to ensure the quality of extracted data. Nevertheless, it is important to acknowledge the limitations of this review. Firstly, the cultural validity of some of the tools used in the reviewed studies remains unclear. It is crucial to conduct validation work to ensure the cultural congruency of tools used in a specific population before their implementation. Secondly, the literature search was constrained by specific research terms and the English language and date range which may have led to the omission of relevant articles.

Furthermore, some studies in this review used small samples and as a result they were unable to provide a definitive answer regarding whether immigration had a positive or negative effect on the experience of IPV among Iranian immigrant women. To overcome such limitations, comprehensive and large-scale studies are necessary to offer a clear and cohesive understanding of the unique experiences, challenges, and barriers faced by Iranian immigrant women in relation to domestic violence. Future research should also include a psychometric evaluation of the tools used and employ mixed method designs to obtain a comprehensive understanding of violence and its impact on the health of immigrant and refugee women.

## Conclusion

While the reviewed studies provide valuable insights into the experiences of IPV among Iranian immigrant women, further research is needed to gain a more comprehensive understanding of the magnitude and impact of IPV in this population. Future studies should explore the barriers faced by these women in accessing legal services and delve deeper into the interplay of individual, socio-cultural, and religious factors that influence their experiences and help seeking behaviours.
